# Facile assembly of flexible, stretchable and attachable symmetric microsupercapacitors with wide working voltage windows and favorable durability

**DOI:** 10.1038/s41378-024-00742-0

**Published:** 2024-08-02

**Authors:** Xiangguang Han, Xiaoyu Wu, Libo Zhao, Min Li, Chen Jia, Zhikang Li, Jiaqi Xie, Guoxi Luo, Ping Yang, Rabah Boukherroub, Yurdanur Türker, Mert Umut Özkaynak, Koray Bahadır Dönmez

**Affiliations:** 1 State Key Laboratory for Manufacturing Systems Engineering, International Joint Laboratory for Micro/Nano Manufacturing and Measurement Technologies, Xi’an Jiaotong University (Yantai) Research Institute for Intelligent Sensing Technology and System, Xi’an Jiaotong University, Xi’an, 710049 China; 2 School of Instrument Science and Technology, Xi’an Jiaotong University, Xi’an, 710049 China; 3 Shandong Laboratory of Advanced Materials and Green Manufacturing at Yantai, Yantai, 264000 China; 4 School of Mechanical Engineering, Xi’an Jiaotong University, Xi’an, Shaanxi 710049 China; 5 Chongqing Key Laboratory of Micro-Nano Systems and Intelligent Sensing, Chongqing Academician Workstation, Chongqing 2011 Collaborative Innovation Center of Micro/Nano Sensing and Intelligent Ecological Internet of Things, Chongqing Technology and Business University, Nan’an District, Chongqing, 400067 China; 6 Univ. Lille, CNRS, Univ. Polytechnique Hauts-de-France, UMR 8520 - IEMN, F-59000 Lille, France; 7 Sabanci University Nanotechnology Research and Application Center (SUNUM), Istanbul, Turkey; 8 Department of Materials Science and Engineering, Istanbul Technical University, Istanbul, Turkey

**Keywords:** Electrical and electronic engineering, Carbon nanotubes and fullerenes

## Abstract

With the increasing development of intelligent robots and wearable electronics, the demand for high-performance flexible energy storage devices is drastically increasing. In this study, flexible symmetric microsupercapacitors (MSCs) that could operate in a wide working voltage window were developed by combining laser-direct-writing graphene (LG) electrodes with a phosphoric acid-nonionic surfactant liquid crystal (PA-NI LC) gel electrolyte. To increase the flexibility and enhance the conformal ability of the MSC devices to anisotropic surfaces, after the interdigitated LG formed on the polyimide (PI) film surface, the devices were further transferred onto a flexible, stretchable and transparent polydimethylsiloxane (PDMS) substrate; this substrate displayed favorable flexibility and mechanical characteristics in the bending test. Furthermore, the electrochemical performances of the symmetric MSCs with various electrode widths (300, 400, 500 and 600 μm) were evaluated. The findings revealed that symmetric MSC devices could operate in a large voltage range (0–1.5 V); additionally, the device with a 300 μm electrode width (MSC-300) exhibited the largest areal capacitance of 2.3 mF cm^−2^ at 0.07 mA cm^−2^ and an areal (volumetric) energy density of 0.72 μWh cm^−^^2^ (0.36 mWh cm^−^^3^) at 55.07 μW cm^−2^ (27.54 mW cm^−3^), along with favorable mechanical and cycling stability. After charging for ~20 s, two MSC-300 devices connected in series could supply energy to a calculator to operate for ~130 s, showing its practical application potential as an energy storage device. Moreover, the device displayed favorable reversibility, stability and durability. After 12 months of aging in air at room temperature, its electrochemical performance was not altered, and after charging-discharging measurements for 5000 cycles at 0.07 mA cm^−2^, ~93.6% of the areal capacitance was still retained; these results demonstrated its practical long-term application potential as an energy storage device.

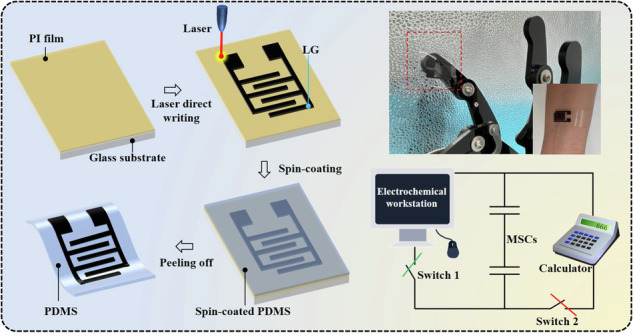

## Introduction

With the arrival of the intelligent era, the demands for intelligent robots, wearable electronic devices and implantable medical sensors have drastically increased^1–4^. Flexible miniature energy storage devices, such as microsupercapacitors (MSCs) and microbatteries, have played indispensable roles in supplying power for various components^5–8^. Among these flexible energy storage devices, MSCs have been recognized for their various beneficial features, including satisfactory power density, prompt charging/discharging time, long lifespan, and high safety^9–11^. However, the drawback of low energy density has seriously hindered their application. Therefore, efforts have been devoted to improving the capacitance or broadening the operating window to obtain a high energy density since the energy density is directly correlated with the capacitance and the square of the working voltage window^12,13^.

Assembling hybrid MSCs is an available strategy for broadening the operating voltage window to increase the energy density. However, these devices have shorter cycling times, higher costs and more complex fabrication processes. Thus, symmetric MSC devices with long lifespans, low costs and simple fabrication processes need to be prepared. For example, Hong et al. successfully fabricated stretchable symmetric MSC devices based on multi-walled carbon nanotubes (MWNT)/Mn_3_O_4_ electrodes and a PVA-H_3_PO_4_ electrolyte and demonstrated an areal capacitance of 0.63 mF cm^−^^2^ in a 0–0.8 V voltage window^14^. Kim et al. prepared a stretchable microsupercapacitor array (3 × 3 array) with planar SWCNT electrodes and an ionic liquid-based triblock copolymer electrolyte and recorded a capacitance of ∼100 μF at a scan rate of 0.5 V s^−^^1^ in a voltage range of 0–3 V^15^. After the laser writing method was first applied to produce graphene-based MSCs on polyimide films, subsequent studies have made substantial progress^16–19^. Along these lines, Pietro et al. designed a laser-induced graphene (LIG)-based MSC device, demonstrating a 0–0.8 V working voltage window with the largest areal capacitance of 287 µF cm^−^^2^ at 2.5 µA cm^−2^ ^20^. Yeong et al. fabricated reduced graphene oxide electrodes using an ultrashort-pulse laser for flexible microsupercapacitors, which could operate in a 0–1 V voltage window and exhibited a maximum energy density of 1.08 mWh cm^−3^ ^21^. Yannik et al. produced interdigital MSC devices with liquid carbon precursors by a one-step printing method; these devices operated in a 0–1 V voltage window and displayed a maximum energy density of 0.3 mWh cm^−3^ ^22^. Although these examples have demonstrated the effectiveness of MSCs, efforts to broaden the device operating voltage window warrant additional research to further enhance the electrochemical performance of symmetric MSC devices.

In this study, flexible symmetric MSCs were prepared by a laser direct writing approach, forming interdigitated LG electrodes with different widths (300, 400, 500 and 600 μm). After being transferred onto a polydimethylsiloxane (PDMS) substrate and using a PA-NI LC gel electrolyte, the symmetric LG-based MSC devices exhibited a wide operating voltage window in the range of 0–1.5 V, with 2.3 mF cm^−2^ at 0.07 mA cm^−2^ as the maximum areal capacitance and 0.72 μWh cm^−2^ (0.36 mWh cm^−3^) at 55.07 μW cm^−2^ (27.54 mW cm^−3^) as the maximum areal (volumetric) energy density. However, when the devices functioned in the 0–1.2 V range, the values decreased to 0.44 μWh cm^−2^ (0.22 mWh cm^−3^) at 12.18 μW cm^−2^ (6.09 mW cm^−3^); this result revealed the importance of broadening the operating voltage window. To assess their practical application as energy storage and supply devices, two MSC-300 (300 μm wide) devices were connected in series to supply sufficient energy to operate for ~130 s. Additionally, the as-assembled MSC devices displayed good mechanical stability with 98.3% capacitance retention after 1000 bending cycles and favorable cycling stability with 95.3% capacitance retention after 5000 cycles, indicating their potential for flexible electronics.

## Experimental section

### Assembly of the LG/LG symmetric MSC devices

To fabricate the MSC devices, a universal laser system with a CO_2_ laser light source (VLS2.30DT, *λ* = 10.6 μm) was applied to in situ produce interdigitated LG electrodes with different widths (300, 400, 500 and 600 μm) directly on the surface of a polyimide (PI) film. The power of the laser was set to 4.5 W with a scan rate of 10 cm s^−1^. To design the flexible MSC devices, transfer printing technology was utilized. A mixture of the PDMS prepolymer and curing agent (10:1 by volume) was spin-coated on the LG electrode surface. The speed was set at 500 rpm min^−1^, and the entire process lasted for 30 s. Then, the sample was dried for 2 h at 70 °C. Subsequently, the PDMS film with the LG electrodes was peeled off from the uncarbonized PI film. After the PA-NI LC gel electrolyte was added dropwise (the preparation process is described in detail in the Supplementary Information file), the MSC device was packaged with a thin PDMS film that was previously treated with oxygen plasma for 60 s. The as-obtained devices were named MSC-300, MSC-400, MSC-500 and MSC-600 for the widths of 300, 400, 500 and 600 μm, respectively.

## Results and discussion

**Fig. 1:**
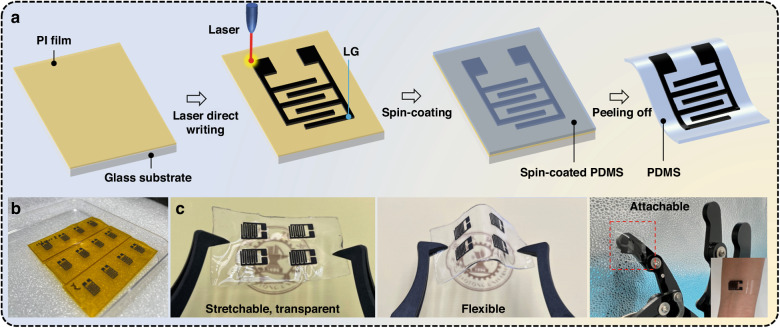
Preparation of the LG-based symmetric MSC devices. **a** Diagrammatic sketch of the preparation process. **b** Photographs of LG-based MSC electrodes after the laser-induced process. **c** Photographs of the as-obtained flexible MSCs

To obtain LG-based MSC devices, as schematically illustrated in Fig. 1a, four steps were conducted: (i) the PI film was tightly attached to the glass sheet surface; (ii) the universal laser system with a CO_2_ laser light source (VLS2.30DT, *λ* = 10.6 μm) was used to directly write the interdigitated LG electrodes on the PI surface using a 4.5 W power and a 10 cm s^−1^ scan rate, as displayed in Fig. 1b; (iii) the PDMS prepolymer and curing agent (10:1 by volume) were mixed together, the mixture was left in the refrigerator overnight, and then the mixture was overlaid on the LG electrode surface and spin-coated (500 rpm min^−^^1^ for 30 s); and (iv) the PDMS film with LG electrodes was peeled off after drying at 70 °C for 2 h and formed a transparent, flexible, stretchable and attachable MSC device, as exhibited in Fig. 1c, indicating promising application potential in wearable electronic devices.

**Fig. 2:**
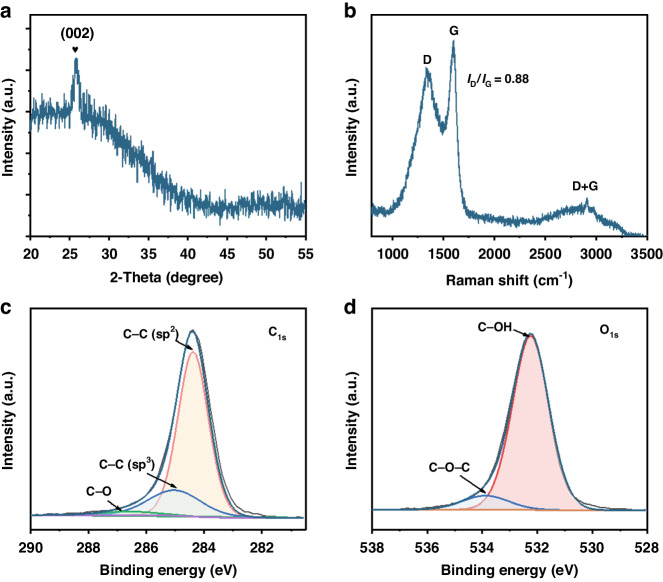
Characterization of the LG electrode. **a** XRD and **b** Raman spectra and **c** C_1s_ and **d** O_1s_ core-level XPS spectra

X-ray diffraction (XRD) measurements were conducted to determine the chemical composition and crystallinity of the as-fabricated MSC electrode. As depicted in Fig. 2a, the typical (002) diffraction plane for the LG electrode appeared at ~25.8°; this result indicated a high degree of graphitization^23^. Raman spectroscopy was carried out to evaluate the structural state of the carbon materials. In Fig. 2b, evident typical peaks appeared at ~1331.9 and 1601.5 cm^−1^ and were ascribed to the D and G bands, respectively. The intensity ratio between the D and G bands (*I*_D_/*I*_G_) was determined to be ~0.88 for the as-prepared LG electrode; thus, the material possessed relatively small structural defects and a high degree of graphitization^24^, which was consistent with the XRD results. The D + G peak at ~2908.4 cm^−^^1^ was likely caused by the defect activation phenomenon^25^. Furthermore, X-ray photoelectron spectroscopy (XPS) measurements were performed to explore the chemical composition of the LG electrode. The C_1s_ core level spectrum (Fig. 2c) consisted of C-C (sp^2^), C-C (sp^3^), and C-O bonds located at 284.3, 284.7 and 286.7 eV, respectively^26^. The O_1s_ core level spectrum in Fig. 2d was fitted with two bands at 532.3 and 533.9 eV, corresponding to C-OH and C-O-C bonds, respectively ^1^.

**Fig. 3:**
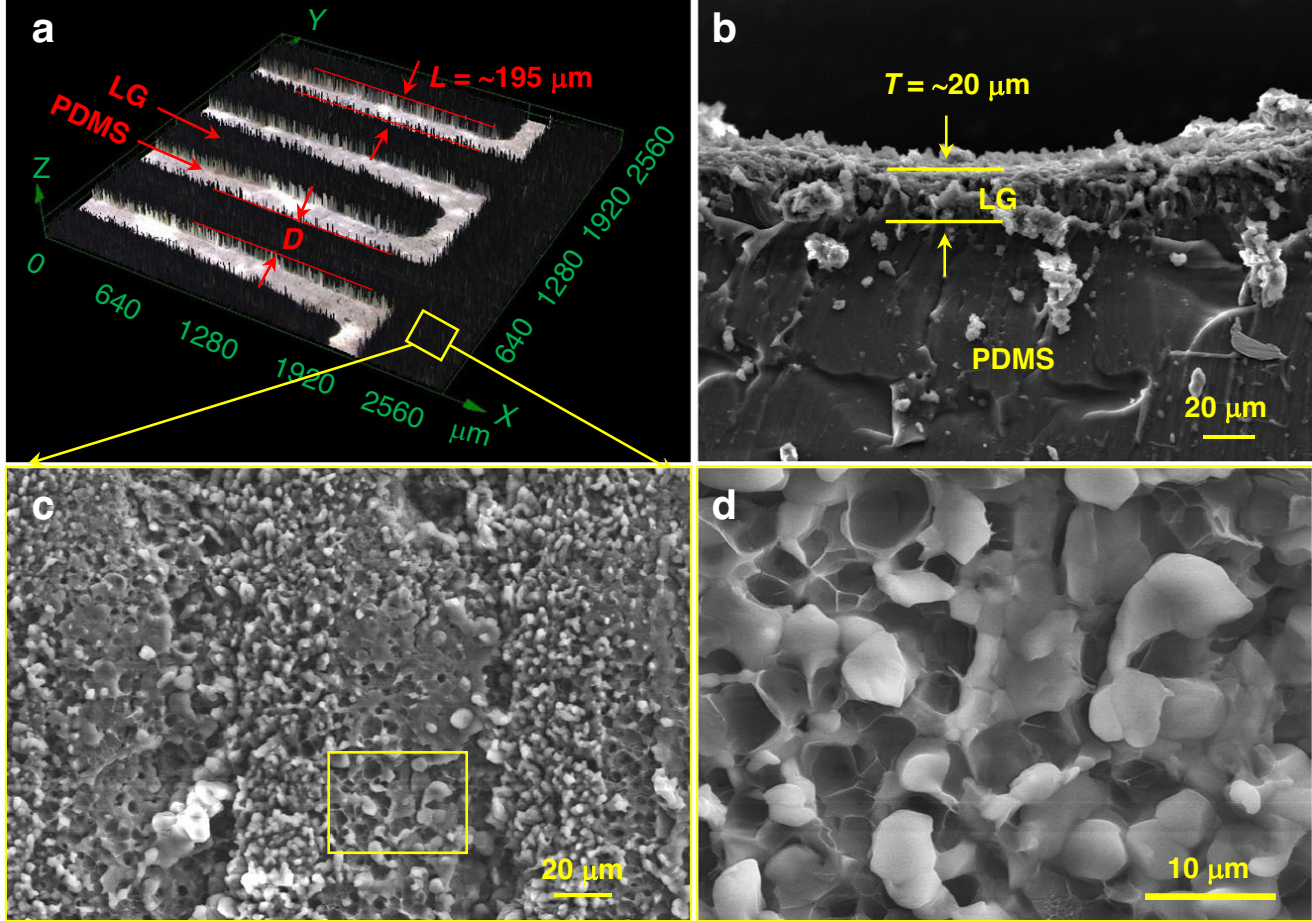
Characterization of the LG-based symmetric MSC electrode. **a** LCSM image. **b** Cross-sectional and **c**, **d** top-view SEM images

Furthermore, the morphology of the LG electrodes was investigated. Figure 3a displays a laser confocal scanning microscopy (LCSM) image of the interdigitated LG electrodes. The black region is the LG material, and the bright region is the space between the two LG electrode fingers. As shown in the image, the spacing distance was ~195 μm. The electrode widths (D) were 300, 400, 500 or 600 μm to explore the effect of the structural parameters on the performance of the MSC devices to optimize their structure. Moreover, the cross-sectional morphology was observed by scanning electron microscopy (SEM), as depicted in Fig. 3b. The thickness of the electrode (*T*) was obtained and measured to be ~20 μm. The surface morphology was also characterized and exhibited a bumpy appearance with numerous micropores (Fig. 3c, d).

**Fig. 4:**
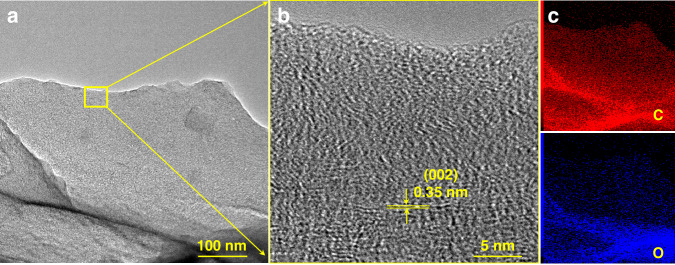
Characterization of the LG electrode. **a**, **b** TEM images and **c** EDS elemental mapping images

Furthermore, transmission electron microscopy (TEM) was performed. The high-resolution TEM image (Fig. 4b), which was selected from Fig. 4a, revealed a 0.35 nm interlayer distance; this could be assigned to the typical (002) plane of the LG electrode^26^. Moreover, Fig. 4c shows the EDS elemental mapping images; here, C and O were present with a uniform distribution in the LG electrode, which was consistent with the XPS results in Fig. 2c, d.

**Fig. 5:**
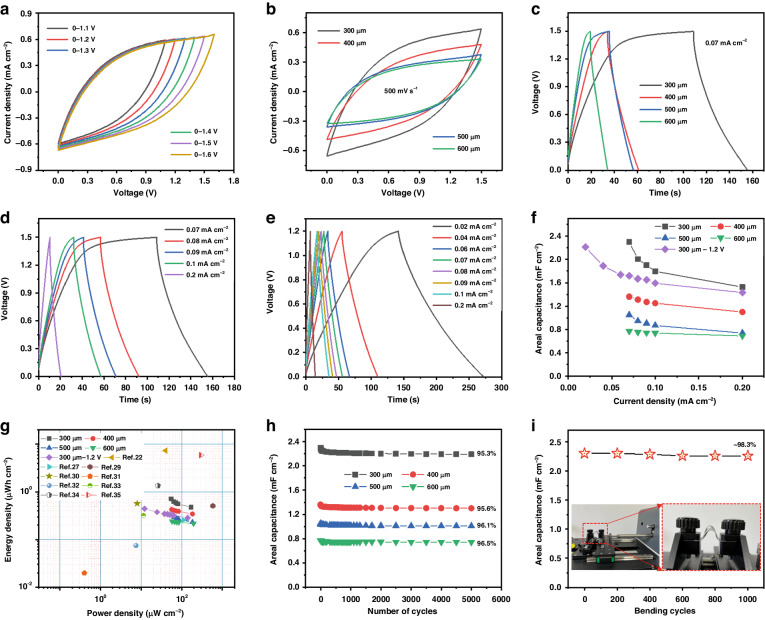
Electrochemical measurements of the symmetric LG-based MSCs with the PA-NI LC gel electrolyte. **a** CV curves of the MSC-300 device recorded at various voltage windows at 500 mV s^−1^; (**b**) CV profiles at 500 mV s^−1^; **c** GCD plots at 0.07 mA cm^−2^ of different MSC devices; GCD curves of the MSC-300 device in the voltage window of **d** 0–1.5 V and **e** 0–1.2 V acquired at various current densities; **f** areal capacitance; **g** Ragone plots; **h** cycling stability; **i** bending test result at 0.07 mA cm^−2^

To investigate and assess the influence of different electrode widths on the electrochemical performance of symmetric LG-based MSCs, various electrochemical tests were carried out. As illustrated in Fig. 5a and Fig. S1, CV measurements of the symmetric MSC devices were conducted in the 0–1.6 V potential range with the PA-NI LC gel electrolyte. Considering the safety and damage, the final working window was selected as 0–1.5 V. As shown in Fig. 5b, the MSC device with a 300-μm electrode width exhibited the largest current density among the four devices, indicating the highest areal capacitance. The same result was obtained from the GCD measurements (0–1.5 V), as shown in Fig. 5c. These results demonstrated that the MSC device with a 300 μm electrode width exhibited the longest discharge time, with the greatest areal capacitance. The corresponding values were determined using Eq. (S1) and the GCD results (Fig. 5d and Fig. S2) and are shown in Fig. 5f. The largest areal capacitance value was obtained for the MSC-300 device and was 2.3 mF cm^−^^2^ at 0.07 mA cm^−2^; this result was likely caused by its smallest intrinsic resistance and charge transfer values among all the devices (Fig. S3 and Table S1). Moreover, to demonstrate the great advantage of the wide working voltage window, a GCD plot between 0 and 1.2 V was also recorded and is displayed in Fig. 5d.

As shown in Fig. 5f, even though the symmetric MSC device could work at lower current densities, lower areal capacitances (2.2 mF cm^−^^2^ at 0.02 mA cm^−2^) and reduced energy and power densities were recorded (Fig. 5g). Among all tested devices, the symmetric MSC-300 device operating in the 0–1.5 V potential range had the largest energy and power density of 0.72 μWh cm^−2^ (0.36 mWh cm^-3^) at 55.07 μW cm^−^^2^ (27.54 mW cm^−3^) according to Eqs. S2–S5; these values were also larger than those of 0.44 μWh cm^−^^2^ (0.22 mWh cm^−3^) at 12.18 μW cm^−^^2^ (6.09 mW cm^−3^) for the same device in the 0–1.2 V range. These results showed the importance of broadening the working voltage window. The largest energy density obtained in this work was comparable to or greater than those reported in several related studies (Table S2); these include the symmetric O/N/S co-doped graphene MSC^11^, symmetric sucrose-derived carbon MSC^22^, symmetric laser-induced graphene MSC^27^, symmetric rGO/CNT MSC^28^, symmetric Ox-SWCNT-MSC-IPL MSC^29^, asymmetric MnO_2_//OLC MSC^30^, asymmetric EG20L//nGO20L MSC^31^, symmetric EEG MSC^32^, symmetric extrusion-printed MXene MSC^33^, symmetric graphene-CNT composite MSC^34^, and symmetric graphene-based MSC^35^.

A stability test was conducted by repeating the charging-discharging measurements for 5000 cycles at 0.07 mA cm^−2^ (Fig. 5h). The as-assembled LG-based devices showed good cycling stability, with 95.3–96.5% of the areal capacitance retained; these results indicated the good reversibility and stability of the LG electrodes. To elucidate the mechanical properties of the flexible and stretchable symmetric MSC device, a tensile test system (FlexTest-S-P2) was utilized. The device was bent at 90° and stretched under 100% stretch strain, and ~98.3% and 66.1% of the original areal capacitance values at 0.07 mA· cm^-2^ were preserved after 1000 cycles of bending and stretching, respectively (Fig. 5i and Fig. S4); these results indicated good flexibility, stretchability and mechanical characteristics of our MSC device.

**Fig. 6:**
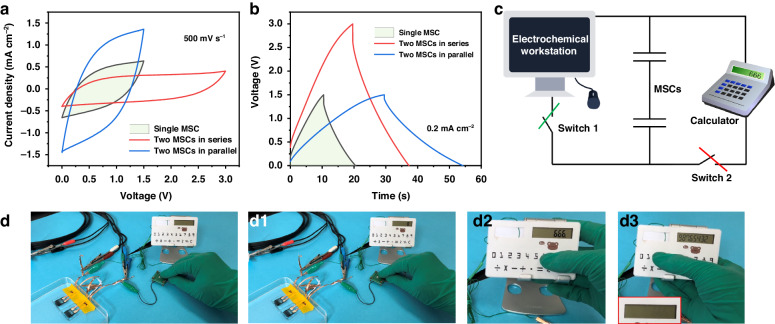
Practical application tests. **a** CV curves at 500 mV s^−^^1^. **b** GCD plots at 0.2 mA cm^−2^ for the MSC-300 device. **c** Schematic equivalent circuit diagram and **d** photographs of the application test system

After in-depth investigation of the electrochemical performance of the MSC-300 device, its practical application ability was further evaluated. The CV (Fig. 6a) and GCD (Fig. 6b) curves revealed that the performance could be improved by connecting these MSC devices in series or parallel; in particular, two MSC-300 devices connected in series could work normally in a voltage range of 0–3 V. Consequently, the two devices were charged by an electrochemical workstation (switch 1 was on, switch 2 was off) and further applied to supply power to an 8-bit display calculator (1.4 V) (switch 1 was off, switch 2 was on). The corresponding schematic equivalent circuit diagram and photographs are illustrated in Fig. 6c, d. At the moment when the voltage was charged to 3 V, switch 2 was turned on (Fig. 6d1), and the calculator worked normally, displaying the number ‘0’. Moreover, this value could be also randomly changed to ‘666’ (Fig. 6d2) and ‘98765432’ (Fig. 6d3) the working process. Notably, the charging time was ~20 s, and the MSCs could supply energy to the calculator to operate for ~130 s until it turned dark, as shown in the inset image in Fig. 6d3; these results indicated promising application potential of these devices.

**Fig. 7:**
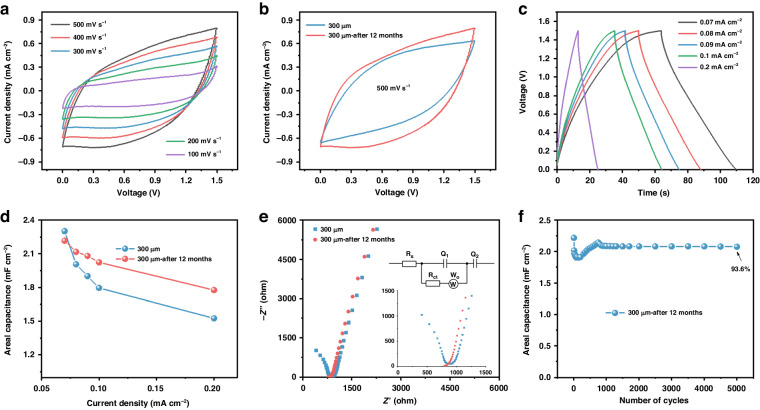
Electrochemical measurements of the MSC-300 device after 12 months of aging in air. **a** CV curves at various scan rates. **b** CV curves at 500 mV s^−^^1^. **c** GCD profiles at various current densities. **d** Areal capacitance. **e** Electrochemical impedance spectra. **f** Cycling stability

Consequently, the aging ability of the as-assembled MSCs to function as efficient energy storage devices for long-term applications was examined. After 12 months of aging in air at room temperature, the electrochemical performance of the same MSC-300 device was continuously evaluated, and the results are shown in Fig. 7. The CV curves in Fig. 7a indicated similar shapes to the results measured using the initial device (Fig. 5a, b), and more specifically, the current density even slightly increased at 500 mV s^−1^, as depicted in Fig. 7b; these results indicated a higher areal capacitance. Moreover, GCD measurements at various current densities were also performed (Fig. 7c), and the corresponding areal capacitance values were calculated and are displayed in Fig. 7d. The areal capacitance was slightly greater than that recorded using the fresh device, which was consistent with the CV results in Fig. 7b. This result could be ascribed to a decrease in the charge transfer resistance, as evidenced by the electrochemical impedance data (Fig. 7e and Table S1). Furthermore, the reversibility and stability of the MSC-300 device after 12 months of aging in air were recorded by repeating the charge‒discharge measurements for 5000 cycles at 0.07 mA cm^−^^2^, as shown in Fig. 7f. Approximately 93.6% of the areal capacitance was retained. The above aging test results revealed the favorable reversibility, stability and durability of the as-fabricated symmetric MSC devices for long-term applications, further confirming their potential as energy storage devices.

## Conclusion

In summary, interdigitated graphene electrodes of symmetric MSCs were prepared by a facile and time-saving laser fabrication method. With the assistance of the PA-NI LC gel electrolyte, the symmetric MSC devices exhibited a large operating voltage window, reaching 1.5 V; this was a significant improvement for symmetric devices. The influence of the electrode width (300, 400, 500 and 600 μm) on the electrochemical performance of the devices was explored to achieve structure optimization; the results revealed that the MSC-300 device with the smallest electrode width possessed the largest areal capacitance and areal (volumetric) energy density of 0.72 μWh cm^−2^ (0.36 mWh cm^−3^) at 55.07 μW cm^−2^ (27.54 mW cm^−^^3^), respectively; these values were greater than the values of 0.44 μWh cm^−2^ (0.22 mWh cm^−3^) at 12.18 μW cm^−2^ (6.09 mW cm^−3^), respectively, for the same device recorded in the 0–1.2 V voltage range. The practical application ability of the devices was assessed, and the device could supply energy to a calculator to operate for ~130 s with a short charging time (~20 s). Furthermore, the MSC device showed favorable cycling stability, durability and good mechanical stability. All findings demonstrated the promising future of the as-fabricated MSC devices as energy storage devices.

### Supplementary information

Revised Supplementary Materials
