# Supplementary material for: Facile assembly of flexible, stretchable and attachable symmetric microsupercapacitors with wide working voltage windows and favorable durability
Source: Microsyst Nanoeng. 2024 Aug 2;10:107. doi: 10.1038/s41378-024-00742-0 (PMC11294472; doi:10.1038/s41378-024-00742-0)
Supplement: Supplementary file 1 — Revised Supplementary Materials [file 41378_2024_742_MOESM1_ESM.docx]

**Facile Assembly of Flexible, Stretchable and Attachable Symmetric Micro-Supercapacitors with Wide Working Voltage Window and Favorable Durability**

Xiangguang Han^a, b, d, #^, Xiaoyu Wu^c, #^, Libo Zhao^a, b, d, h^, Min Li^a, b, d,^ *, Chen Jia^a, b,^ *, Zhikang Li^a, b, d^, Jiaqi Xie^a, c^, Guoxi Luo^a, b, d^*, Ping Yang^a, c^, Rabah Boukherroub^e^, Yurdanur Türker^f^, Mert Umut Özkaynak^f, g^, Koray Bahadır Dönmez^f^

*^a^* *State Key Laboratory for Manufacturing Systems Engineering, International Joint Laboratory for Micro/Nano Manufacturing and Measurement Technologies, Xi’an Jiaotong University (Yantai) Research Institute for Intelligent Sensing Technology and System, Xi’an Jiaotong University, Xi’an 710049, China*

*^b^ School of Instrument Science and Techonology, Xi’an Jiaotong University, Xi’an, 710049, China*

*^c^School of Mechanical Engineering, Xi'an Jiaotong University, Xi'an, Shaanxi, China, 710049*

*^d^ Shandong Laboratory of Advanced Materials and Green Manufacturing at Yantai, Yantai, 264000, China*

*^e^ Univ. Lille, CNRS, Univ. Polytechnique Hauts-de-France, UMR 8520 - IEMN, F-59000 Lille, France*

*^f^ Sabanci University Nanotechnology Research and Application Center (SUNUM), Istanbul, Turkey*

*^g^ Department of Materials Science and Engineering, Istanbul Technical University, Istanbul, Turkey*

*^h^ Chongqing Key Laboratory of Micro-Nano Systems and Intelligent Sensing, Chongqing Academician Workstation, Chongqing 2011 Collaborative Innovation Center of Micro/Nano Sensing and Intelligent Ecological Internet of Things, Chongqing Technology and Business University, Nan’an District, Chongqing 400067, China*

*To whom correspondence should be addressed: Min LI (limin@xjtu.edu.cn), Chen Jia (jiachen0224@stu.xjtu.edu.cn), Guoxi Luo (luoguoxi@mail.xjtu.edu.cn).

^#^Equal contribution.

**EXPERIMENTAL SECTION**

**Materials and reagents**. Phosphoric acid (H_3_PO_4_.H_2_O, PA) with a purity of 99.99% was obtained from Sigma-Aldrich (China). Lauryl ether 23 was procured from JK Scientific (Turkey) with a purity of 100% and used as a non-ionic surfactant (NI). Polyimide (PI) film was purchased from Zhongshan Chenxi Technology Co., Ltd (China). Polydimethylsiloxane (PDMS) was bought from Sigma-Aldrich (China).

**Preparation of the** **PA-NI LC gel electrolyte**. The preparation of the phosphoric acid-non-ionic surfactant liquid crystal (PA-NI LC) gel electrolyte was performed according to our previous work.^S1^ Firstly, 1.0 g of lauryl ether 23 was heated at its melting point of 50 ^o^C. Then, it was mixed with 7.6 g of phosphoric acid (PA, 85.0% wt/wt) in a 20 mL glass vial. This mixture was homogenized at 80 ^o^C for 15 min to obtain the LC gel phases.

**Characterization**. The crystal structure was characterized by X-ray diffraction (XRD; D8 Advanced, Bruker) using Cu Kα radiation in the *2θ* range of 10° to 80°. Raman spectroscopy measurements were performed on a Laser Raman spectrometer (HORIBA) using a 532-nm laser diode as excitation source. Visible light was focused by a 50× objective. The scattered light was collected by the same objective in backscattering configuration, dispersed by an 800 mm focal length monochromator and detected by a CCD camera. X-ray photoelectron spectra (XPS) were recorded on a photoelectron spectrometer (ESCALAB Xiþ, ThermoFisher Scientific) with Al Kα as the X-ray source. The laser confocal scanning microscope (LCSM) images were obtained by using OLS4000 microscope. SEM images were acquired to observe the morphology and structure of as-prepared samples using a field-emission scanning electron microscope (FESEM; Gemini SEM 500, Zeiss, 15 kV). Transmission electron microscopy (TEM) and high-resolution TEM (HRTEM) micrographs, and selected area electron diffraction (SAED) were recorded with a transmission electron microscope (TEM; JEM-F200, JEOL) operating at 200 kV. Elemental characterization was conducted with an energy dispersive X-ray spectrometer (EDS) through areal elemental mapping and analysis.

**Electrochemical measurements**. Cyclic voltammetry (CV) curves were collected at different scan rates (100 - 500 mV·s^-1^). Galvanostatic charge-discharge (GCD) measurements were recorded at various current densities ranging from 0.08 to 0.4 mA·cm^-2^. Electrochemical impedance spectroscopy (EIS) was performed in the frequency range from 10^-2^ Hz to 10^5^ Hz.

**Electrochemical measurements**. The Gamry Reference 600+ electrochemical workstation was applied in this work to perform the electrochemical measurements, including CV, GCD and electrochemical impedance spectroscopy (EIS) tests.

The areal capacitance was calculated from the GCD profiles using equation (S1):

$$\text{C}\text{ (mF}\text{⋅}\text{cm}^{\text{-2}}\text{)=}\frac{\text{I}\text{×}\text{Δt}}{\text{A×}\text{ΔV}} \text{(S1)}$$

where *C* (mF·cm^-2^) is the areal capacitance, *I* (A) is the discharge current, *Δt* (s) represents the discharge time, *A* (cm^-2^) is the whole footprint area of active interdigitated electrodes, and *ΔV* (V) is the discharge voltage window.

The areal energy density (*E*, μWh⋅cm^-2^), areal power density (*P*, μW⋅cm^-2^), volumetric energy density (*E*, mWh⋅cm^-3^) and volumetric power density (*P*, mW⋅cm^-3^) of the MSC devices were calculated using equations (S2-5), respectively:

$$\text{E }\text{(}\text{μWh}\text{}\text{cm}\text{-2}\text{)}\text{=}\frac{\text{0.5}\text{C}\text{×∆}\text{V}^{\text{2}}}{\text{3.6}}\text{ }\text{(S2)}$$

$$\text{P}\text{ }\text{(}\text{μW}\text{}\text{cm}\text{-2}\text{)}\text{=}\frac{\text{E×}\text{3600}}{\text{∆t}}\text{ }\text{(S3)}$$

$$\text{E }\text{(}\text{mWh}\text{}\text{cm}\text{-3}\text{)}\text{=}\frac{\text{0.5}\text{C}\text{×∆}\text{V}^{\text{2}}}{\text{3.6}\text{×T}}\text{ }\text{(S4)}$$

$$\text{P}\text{ }\text{(}\text{mW}\text{}\text{cm}\text{-3}\text{)}\text{=}\frac{\text{E×}\text{3600}}{\text{∆t}\text{×T}}\text{ }\text{(S5)}$$

where *C* (mF⋅cm^-2^) stands for the areal capacitance, ∆*V* (V) is the potential window of discharge, *Δt* (s) is the discharge time, *T* (cm) is the thickness of the electrode.


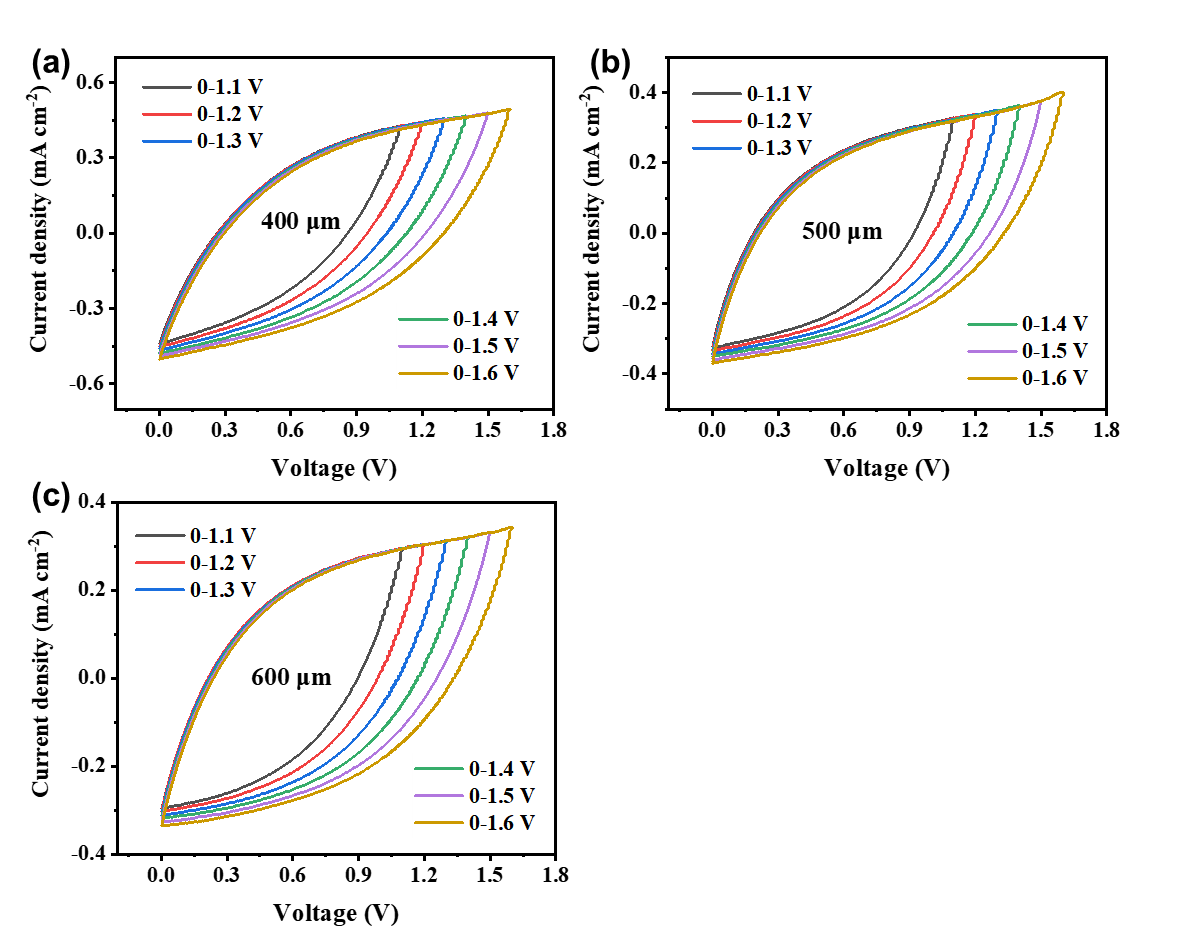


**Figure S1.** CV plots of LG-based MSCs devices with electrode widths of (**a**) 400 μm, (**b**) 500 μm and (**c**) 600 μm measured in PA-NI LC gel electrolyte at 500 mV·s^-1^.


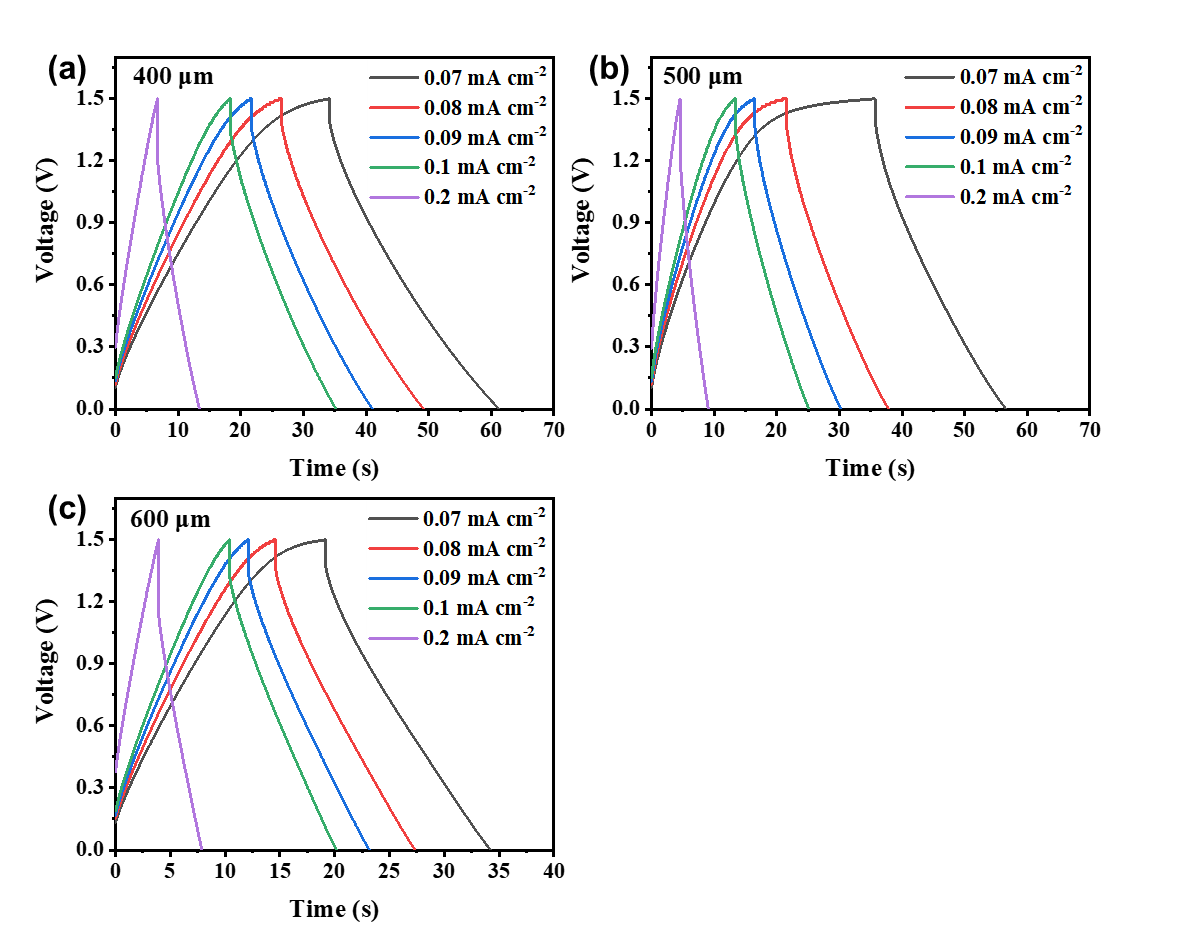


**Figure S2.** GCD profiles of LG-based MSCs devices with electrode widths of (**a**) 400 μm, (**b**) 500 μm and (**c**) 600 μm measured in PA-NI LC gel electrolyte at various current densities.


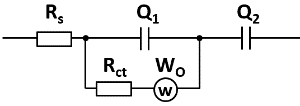


**Figure S3.** Electrochemical impedance spectra of LG-based MSCs with PA-NI LC gel electrolyte. The insert in upper-right corner is the corresponding equivalent circuit of the EIS results. The insert in lower-right corner is the magnified spectra.

**Figure S4.** Stretch stability test of MSC-300 device under 100% stretch strain after different stretching cycles.

**Table S1.** Resistance values of as-prepared MSCs.

| **Electrode width (μm)** | **Intrinsic resistance**  **(*R_s_*) /** **ohm cm^-2^** | **Charge transfer resistance (*R_ct_*) / ohm cm^-2^** |
| --- | --- | --- |
| 300 | 0.03 | 1121.0 |
| 400 | 0.07 | 1143.2 |
| 500 | 0.13 | 1163.2 |
| 600 | 0.18 | 1256.0 |
| 300-after 12 months | 787.5 | 309.4 |

**Table S2.** Energy densities comparison of different MSCs.

| **Device** | **Energy density** | **Power density** |
| --- | --- | --- |
| Symmetric sucrose-derived carbon MSC^11^ | 0.3 mWh⋅cm^-3^ | 100 mW⋅cm^-3^ |
| Symmetric O/N/S co-doped graphene MSC^22^ | 7.3 µWh/cm^2^ | 40 μW⋅cm^-2^ |
| Symmetric laser-induced graphene MSC^27^ | 0.256 µWh/cm^2^ | 110 μW/cm^2^ |
| Symmetric rGO/CNT MSC^28^ | 0.31 μWh⋅cm^-3^ | 89.5 mW⋅cm^-3^ |
| Symmetric Ox-SWCNT-MSC-IPL MSC^29^ | 0.51 μWh⋅cm^-2^ | 590 μW⋅cm^-2^ |
| Asymmetric MnO_2_//OLC MSC^30^ | 0.57 μWh⋅cm^-2^ | 8 μW⋅cm^-2^ |
| Asymmetric EG20L//nGO20L MSC^31^ | 0.02 μWh⋅cm^-2^ | 0.4 μW⋅cm^-2^ |
| Symmetric EEG MSC^32^ | 0.075 μWh⋅cm^-2^ | 7.5 μW⋅cm^-2^ |
| Symmetric extrusion-printed MXene MSC^33^ | 0.32 μWh⋅cm^-2^ | 11.4 μW⋅cm^-2^ |
| Symmetric graphene-CNT composite MSC^34^ | 1.36 μWh⋅cm^-2^ | 26 μW⋅cm^-2^ |
| Symmetric graphene-based MSC^35^ | 5.9 μWh⋅cm^-2^ | 300 μW⋅cm^-2^ |
| **This work** | 0.72 μWh⋅cm^-2^  (0.36 mWh⋅cm^-3^) | 55.07 μW⋅cm^-2^  (27.54 mW⋅cm^-3^) |

**Reference**

S1. Li, M. *et al.* An efficient cobalt-nickel phosphate positive electrode for high-performance hybrid microsupercapacitors. *J. Energy Storage* **64**, 107144 (2023).
